# Dynamics of the nasopharyngeal microbiome of apparently healthy calves and those with clinical symptoms of bovine respiratory disease from disease diagnosis to recovery

**DOI:** 10.3389/fvets.2023.1297158

**Published:** 2023-11-16

**Authors:** Ruth Eunice Centeno-Martinez, Rebecca N. Klopp, Jennifer Koziol, Jacquelyn P. Boerman, Timothy A. Johnson

**Affiliations:** ^1^Department of Animal Science, Purdue University, West Lafayette, IN, United States; ^2^School of Veterinary Medicine, Texas Tech University, Amarillo, TX, United States

**Keywords:** calf, *Lactobacillus*, community succession, bovine respiratory disease, antibiotics

## Abstract

**Introduction:**

Bovine respiratory disease (BRD) is a multifactorial disease complex in which bacteria in the upper respiratory tract play an important role in disease development. Previous studies have related the presence of four BRD-pathobionts (*Mycoplasma bovis*, *Histophilus somni*, *Pasteurella multocida*, and *Mannheimia haemolytica*) in the upper respiratory tract to BRD incidence and mortalities in the dairy and beef cattle industry, but these studies typically only use one time point to compare the abundance of BRD-pathobionts between apparently healthy and BRD-affected cattle. The objective of this study was to characterize the longitudinal development of the nasopharyngeal (NP) microbiome from apparently healthy calves, and in calves with clinical signs of BRD, the microbiota dynamics from disease diagnosis to recovery.

**Methods:**

Deep nasopharyngeal swabs were taken from all calves immediately after transport (day 0). If a calf was diagnosed with BRD (*n* = 10), it was sampled, treated with florfenicol or tulathromycin, and sampled again 1, 5, and 10 days after antibiotic administration. Otherwise, healthy calves (*n* = 20) were sampled again on days 7 and 14. Bacterial community analysis was performed through 16S rRNA gene amplicon sequencing.

**Results:**

The NP microbiome of the healthy animals remained consistent throughout the study, regardless of time. The NP microbiota beta diversity and community composition was affected by tulathromycin or florfenicol administration. Even though BRD-pathobionts were identified by 16S rRNA gene sequencing in BRD-affected animals, no difference was observed in their relative abundance between the BRD-affected and apparently healthy animals. The abundance of BRD-pathobionts was not predictive of disease development while the relative abundance of BRD pathobionts was unique to each BRD-affected calf. Interestingly, at the end of the study period, the genera *Mycoplasma* was the most abundant genus in the healthy group, while *Lactobacillus* was the most abundant genus in the animals that recovered from BRD.

**Discussion:**

This study highlights that injected antibiotics seem to improve the NP microbiome composition (higher abundance of *Lactobacillus* and lower abundance of *Mycoplasma*), and that the relative abundance of BRD-pathobionts differs between individual calves but is not strongly predictive of BRD clinical signs, indicating that additional factors are likely important in the clinical progression of BRD.

## Introduction

In the dairy and beef cattle industry, the development of bovine respiratory disease (BRD) is an ongoing problem. This disease is caused by bacterial and/or viral infection in the cattle respiratory tract compromising its immune system (1). Cattle affected with BRD can show acute fatal respiratory disease or chronic and prolonged intractable disease (2). Additionally, BRD represents a critical economic problem (3) from which the expenses related to animal death, reduction of feed efficiency, and treatment costs have been estimated to be $800–900 M in the US (4).

The development of BRD is related to and exacerbated by multiple factors such as temperature, stocking density, and transportation stress, as well as from viral and bacterial infections including the presence of BRD-pathobionts *Pasteurella multocida*, *Histophilus somni*, *Mycoplasma bovis*, and *Mannheimia haemolytica* (5, 6). Some combination of these factors affect the host immune system and initiate infection and disease. Previous studies characterized the bacterial community of the upper respiratory tract and quantified the presence of BRD-pathobionts in BRD-affected cattle and healthy pen-mates at a single time point (7–12) or using longitudinal designs to determine the change in the microbiome between both groups before and after transport (13–15) or comparing the change before and after BRD diagnosis (16–18). From the transportation studies, it has been possible to determine that transportation increases BRD development from 16.0% to 82.8% due to a BRD-pathobiont co-infection present in the upper respiratory tract (15) and a dynamic change in the upper respiratory tract (URT) microbiome occurred 2 days after transport (14). Nonetheless, these studies relied on the comparison of only two timepoints, potentially missing the timing of community shifts or other important indicators of how disease spreads between the animals. Currently, intensive efforts have focused on understanding the change in the URT microbiome composition as a means to determine when an animal could be developing BRD. A study compared the change in the nasopharyngeal microbiome of healthy calves, calves with respiratory disease, otitis, or both diseases throughout the first 35 days of life and revealed that the presence of *Mannheimia* and *Mycoplasma* are important pathogens for both otitis and pneumonia development (17). Furthermore, high neonatal bacterial load in the NP is related to pneumonia development. Additionally, the same authors identified that the NP microbiota is affected by time and age, specifically with a decrease in microbial richness from 3 to 14 days of life (17), highlighting the dynamic characteristic of the microbial population.

Understanding the dynamics of the NP microbiome over time is crucial to determine the conditions when BRD-pathobionts display a commensal or pathogenic phenotype. In addition, it is necessary to understand how the NP microbiome changes once animals with BRD clinical signs receive antibiotic treatment. Thus, the objective of this study was to describe the NP microbiome progression of pre-weaned and post-transported Holstein calves during the first 2 weeks of life, and to characterize the calf NP microbiome when diagnosed and treated for BRD. We hypothesized that animals identified with BRD clinical signs will have a higher abundance of BRD-associated pathogens in the NP cavity than animals that remain apparently healthy throughout the study and that antibiotic treatment for the respiratory disease will decrease the NP alpha and beta diversity in BRD-affected animals.

## Materials and methods

### Animals and facility

The study was reviewed and approved by the Purdue University Institutional Animal Care and Use Committee (Protocol #1808001783) and animals utilized for this study were part of a companion study (19). A total of 30 Holstein bull calves, 2–9 days of age, were transported 35 km by trailer to the Purdue University Animal Science Research and Education (ASREC) Dairy Farm. Once at the facility, they were placed in individual hutches with the purpose to prevent any physical interaction between calves. The individual hutches had an outside area (11.5 × 4 ft) and bedded with pine shaving and re-bedded as necessary. Calves were divided into two dietary treatments as described in Klopp et al. (19). Preweaning intake, including milk replacer (MR, pints) and calf starter (g), and the average daily gain (ADG, kg/days) were averaged for the 2 weeks of the study, as stated by Klopp et al. (19).

### Disease diagnosis

Respiratory scores were recorded for each calf throughout the study following Klopp et al. (19) protocol. Respiratory status was evaluated daily. Calves were diagnosed with BRD if they showed 2 or more clinical signs of BRD (ocular or nasal discharge, labored, rapid or raspy breathing, droopy ears, coughing, fever or refused milk replacer), following a modified Wisconsin calf respiratory chart (20). At the end of the study, 20 animals remained healthy and 10 showed signs of respiratory disease.

### Antibiotic treatment protocol

Detection of respiratory illness was identified by the clinical signs such as ocular or nasal discharge, rapid or raspy breathing, cough, fever. Animals diagnosed with BRD were treated with florfenicol (Nuflor, Merck Animal Health, Kenilworth, NJ) or tulathromycin (Draxxin, Zoetis US, Parsippany, NJ). Florfenicol (3 mL/100 lb BW) was administered intramuscularly, in two doses given 48 h apart. Tulathromycin (1.1 mL/100 lb BW) was administered in one subcutaneous injection. By way of background, both tulathromycin and florfenicol inhibit protein synthesis by binding to the 50S ribosomal unit. While tulathromycin has a limited antibacterial spectrum (21), florfenicol is a synthetic broad-spectrum antibiotic (22). Antibiotic treatment selection for our study was based on veterinarian consultation and farm treatment history.

### Cattle NP swab collection and DNA extraction

Nasopharyngeal (NP) swabs were collected from the 30 animals on days 0 (arrival at Purdue University Dairy Farm), 7, and 14 of the study. However, if a calf was treated with an antibiotic for respiratory disease, NP swabs were collected before treatment (day 0, day of diagnosis and treatment) and 1, 5, and 10 days after disease treatment. Thus, for the animals that developed clinical signs of BRD we collected data on day 0, 7 or 14 and when the illness diagnosis was confirmed. Even though NP samples were collected on days 0, 7, and 14 for the BRD-affect calves, these samples were not included in the study due to missing information. At the end of the study, 10 animals were treated for BRD, while 20 did not present clinical signs of BRD. On average, calves were diagnosed with BRD on day 7 of the study (Supplementary Table S2). The day of sampling during the treatment period (days 0, 1, 5, and 10) was used to characterize the NP microbiome relative to clinical sign development and not relative to the age of the animals. The animals treated for respiratory disease in this study will be called BRD-affected animals, whereas animals that were not diagnosed with a respiratory disease during the entire study period will be identified as apparently healthy animals. Samples collected from apparently healthy animals on days 0, 7, and 14 will be referred as the healthy period. However, samples collected from BRD animals on treatment days 0, 1, 5, and 10 will be referred as the treatment period. Nasopharyngeal samples were collected using a double-guarded deep-nasal swab. Before NP swab collection, the calves’ nares were cleaned with paper towel. After collection, swabs were placed into a 15 mL conical tube containing 5 mL of PBS solution. The tube was labeled with the animal ID, pen, and date and transported (in ice) to the lab for further processing.

Nasopharyngeal swabs were processed to extract the bacterial and mucosal content from the tip of the swabs before DNA extraction. The tip of the swabs along with the PBS solution was transferred to a 1.5 mL tube. Then, the tubes were vortexed horizontally 5 times for 30 s; after mixing the content, the tip of the swabs was removed from the tubes and the content was centrifuged for 10 min at 6,000 g. After this, the supernatant was removed from the tubes, and the mucosal pellets were stored at −20°C until processing. DNA was extracted from the mucosal pellet using the MagAttract PowerMicrobiome DNA/RNA EP Kit (Qiagen, Germantown, MD, United States) following the manufacturer’s protocol. After extraction, the DNA quality was measured using Nanodrop 2000/2000c Spectrophotometer and the concentration was measured with Qubit 4 (Thermo Fisher Scientific, PA, United States).

The extracted DNA was used to create a 16S rRNA gene amplicon library. This library was constructed using a barcode-indexed amplification product from the V4 region of the 16S rRNA gene following the protocol described by Kozich et al. (23). PCR amplification was performed using AccuPrime^™^ Pfx SuperMix (Thermo Fisher Scientific, MA, United States) following Kozich et al. (23) protocol. PCR-grade water was used as the negative control and a mock community (20 Strain Even Mix 138 Genomic Material; ATCC^®^ MSA-1002TM) as a positive control. PCR amplification quality was checked via gel electrophoresis. Amplified DNA was normalized using SequalPrep Normalization Kit, and 5 μL of each amplified sample was used to create a pool at an equal molecule ratio. The amplicons were sequenced via Illumina MiSeq Sequences (2 × 250 paired-end) at the Purdue Genomic Core Facility.

### Bioinformatics analysis

Demultiplex reads were analyzed using Quantitative Insight Intro Microbial Ecology (QIIME2 v.2-2020.2). The raw reads were trimmed using DADA2, the denoising step, and the forward sequences were trimmed at positions 17 and 233, whereas the reverse sequences were trimmed at positions 30 and 166; this allowed to have sequences with a quality >Q30. Taxonomy was assigned using SILVA 132, 515F/806 region database. In addition, for five BRD animals, two NP swabs were collected on day 10 (treatment period), one swab was inserted in the right nostril and the second one in the left nostril. These samples were processed separately during sequencing but were then combined during the bioinformatic analysis after rarefaction.

### Mock community, and negative control sequencing analysis

The raw 16S rRNA sequences of the controls including water, mock community, and negative control for the DNA extraction kit, were analyzed separately using QIIME2 v.2-2020.2 as described above. One ASV identified as *Pseudoalteromonas*, was present in almost every sample, but not in the positive and negative controls, with an average relative abundance >40%. Interestingly, this bacterium is mostly found in marine environments (24). Thus, we removed the ASV assigned to *Pseudoalteromonas* from the study because it was considered contamination. After assigning the taxonomy, the genus *Escherichia-Shigella*, *Clostridium sensu stricto* 1, and *Lactobacillus* composed more than 40% of the relative abundance in the water control samples. We then extracted the 16S rRNA sequences of *Escherichia-Shigella*, *Clostridium sensu stricto* 1, and *Lactobacillus* found in the controls (PCR grade water as negative control and the Mock community as the positive control) and performed a multiple sequence alignment with the ASVs assigned to the same genus found in the NP samples. The multiple sequence alignment was performed using the Clustal Omega supported by EMBL’s European Bioinformatics Institute (25, 26). Any ASV that matched 100% between the control and the ones found in the NP samples was selected for further analysis. To determine if the ASVs that matched 100% between the controls and the samples are potential contaminants, the relative abundance of each ASV was calculated for each scenario: relative abundance in the control samples (mock community, water, and DNA extraction negative control) compared with the relative abundance in the NP samples.

This analysis was performed separately for the samples of the healthy and treatment periods. We first determined the abundance of all ASVs classified as *Escherichia-Shigella*, *Clostridium sensu stricto* 1, and *Lactobacillus* (Supplementary Figures S1A,D,G) followed by calculating the relative abundance of the ASVs classified at genus level that matched 100% with ASVs found in the controls (Supplementary Figures S1B,E,H). Then, we plotted the relative abundance of each specific ASVs (Supplementary Figures S1C,F,I). This allowed us to determine how the “potential ASV contaminants” account for the abundance of these genera in the samples. Separately, we calculated the relative abundance of ASVs classified as *Escherichia-Shigella*, *Clostridium sensu stricto* 1, and *Lactobacillus* in the controls (Supplementary Figure S2). Interestingly, in the samples, ASV412 classified as *Escherichia-Shigella* presented high relative abundance in the healthy and sick animals, and it was prominent at all time points (Supplementary Figure S1). No other ASV in the samples was abundant in all samples across time or between healthy and sick animals. The ASV412 matched 100% with ASV1 (in control samples), also classified as *Escherichia-Shigella* (Supplementary Figure S2A). It was possible to observe that ASV1 was also abundant across all the controls (mock community, water, and DNA-negative control). Therefore, due to its high abundance in the samples and similarity with ASV1 present in the controls, the ASV412 was considered a contaminant, and it was removed from the analysis.

After removing potential contaminants (ASVs assigned to *Pseudoalteromonas* and ASV412 classified as *Escherichia-Shigella*), the sequences were then rarified to 1,277 sequences per sample to calculate the alpha and beta diversity. Alpha diversity was estimated using QIIME2 using Observed ASVs, and Faith’s phylogenetic diversity (Faith’s PD). Beta diversity was measured with the Bray–Curtis dissimilarity Index and Weighted UniFrac and plotted as principal coordinate analysis (PCoA) using R v 4.0.3.

### Statistical analysis for 16S rRNA gene sequencing data

Samples belonging to the healthy (*n* = 20, days 0, 7, and 14) and treatment (*n* = 10, days 0, 1, 5, and 10) periods were analyzed separately to measure the effect of time in the cattle’s NP microbiome. In the case of the treatment period which includes the calves diagnosed and treated for BRD, the antibiotic treatment: florfenicol (Nuflor, *n* = 3) or tulathromycin (Draxxin, *n* = 6) was included as a categorical factor. One calf was removed from the dataset due to death days after NP swab collection (day 0). The NP samples used in this study were collected from animals that were part of a nutrition supplementation trial (19). In that study, animals were assigned to two dietary treatments: *Saccharomyces cerevisiae* fermentation products (SCFP) and control (19). We analyzed the effect of dietary treatment in the NP microbiome of healthy and BRD-affected animals and no significant effect (*p* > 0.05) was observed; thus, dietary treatment was not included in the further statistical analysis. Additionally, animal preweaning intake (MR and starter) and ADG was analyzed to confirm disease status.

Alpha diversity metrics (Observed ASVs, and Faith’s PD) were analyzed using a General Linear Mixed Model for repeated measures using the afex package (*p* ≤ 0.05). To analyze the healthy period samples, the day of sampling was included as a continuous fixed factor, whereas for the treatment period, antibiotic (florfenicol and tulathromycin) treatment was also included in the model. The random effect structure to analyze the alpha diversity metric Observed ASVs and Faith’s PD were specified as random intercepts and slopes with correlations for the within factor, day of sampling, and animal ID.

For the analysis of beta diversity, a Permutational Multivariate Analysis of Variance Test (PERMANOVA; *p* ≤ 0.05) from the vegan package was performed using the Bray–Curtis and Weighted UniFrac distances. For the day effect during the healthy and the treatment period, a PERMANOVA analysis was performed with the calf ID specified as the blocking factor using the argument “strata.” To identify if the NP beta diversity of the tulathromycin and florfenicol groups were different, samples were divided based on each day (1, 5, and 10). Samples from day 0 were not considered in this analysis because samples were collected before antibiotic administration. A dispersion test was performed using a Multivariate homogeneity of groups dispersions (variances), betadisper, from the vegan package (27, 28). We then performed an ANOVA for repeated measure analysis to analyze the time effect on the different periods. In this step, we included the average distance from each day and the average distance for each animal across the different time points to account for the within subject error. If the effect of time was significant, we performed a Tukey–Kramer *post hoc* comparison, using *p* ≤ 0.05 as statistical significance.

Additionally, to analyze the change in the NP microbiome through time (healthy and treatment periods), a beta regression was performed (29) using the package “glmmADMB” (30). In this analysis, the relative abundance of ASVs grouped at phylum and genera level was used as an input to create the model and analyzed separately for the two periods, and the day of sampling was included as a continuous factor. In the case of BRD-affected animals, beta regression was performed separately for each of the antibiotic treatments. The random effect structure in all the cases was specified as random intercepts including the day of sampling as within factor and the animal ID. The regression coefficient was used to determine the degree of change for each ASV across time and the *p*-value obtained was adjusted using the Benjamini–Hochberg method. Lastly, to identify the effect of antibiotics on the NP microbiome and recovery, NP swabs collected on day 1 (after antibiotic treatment administration), day 5, and day 10 were compared to day 0 (before antibiotic treatment). This analysis was performed separately for the tulathromycin and florfenicol groups. A similar random effect structure as mentioned before was used for each of the day comparisons. Additionally, the relative abundance of ASVs identified as BRD-pathobionts was calculated on days 0, 1, 5, and 10 for each calf to determine the abundance of these pathobionts on the day of diagnosis and after antibiotic treatment.

Lastly, for the analysis of illness, samples on day 7 (*n* = 11) from the healthy period were compared to samples of sick animals on 0 day (*n* = 8). We decided to utilize samples of the animals that remained apparently healthy animals collected on day 7 because this was also the average day of the study when the BRD-affected animals were identified by the farm personnel and then treated with antibiotics. Additionally, any apparently healthy animal that was treated with antibiotic to treat other disease not related with respiratory disease was not included in the analysis. The bacterial richness (Observed ASVs), and Faith’s PD phylogenetic diversity were analyzed using a *t*-test. The sickness effect was also analyzed for the Beta diversity using a PERMANOVA test (*p* ≤ 0.05) along with a dispersion test. A negative binomial distributions method, DESeq, was used to identify differentially abundant taxa between healthy and sick animals.

## Results

### Animal performance data

Milk replacer intake and ADG was averaged from day 0 to 14 of the study for each individual animal. No significant difference was observed in the ADG between the healthy and sick group. Animals classified as healthy (167.3 pints ± 1.60) had significantly higher MR intake than animals identified with respiratory disease, 166.0 pints ± 1.69 (*p* = 0.01, Supplementary Figure S3), which can be considered as another illness indicator.

### Bovine nasopharyngeal microbiome alpha diversity

A total of 2,765,374 sequences were identified before the denoising step (DADA2) and 2,290,746 sequences remained after denoising. After removing ASV assigned to *Pseudoalteromonas* and ASV412 assigned to *Escherichia-Shigella*, and combining the samples belonging to the same animal, the samples were rarified to 1,277 sequences per sample. In this step, 11 samples were lost due to low sequence count, containing *Pseudoalteromonas* sequences or due to combination of sample replicates. After rarefying the samples, a total of 3,859 ASVs were used for the alpha and beta analysis. In this study, the effect of time in the NP microbiome was analyzed separately for apparently healthy animals from the BRD-affected group. During the healthy period (days 0, 7, and 14), no significant effect was observed on the Observed ASVs and in the bacterial phylogenetic diversity determined by Faith’s PD as time passed (*p* ≥ 0.05). When comparing the NP microbiome of apparently healthy (7 days, healthy period) and BRD-affected animals (0 day, treatment period), no sickness effect (*p* ≥ 0.05) was observed between the two groups in Observed ASVs or in Faith’s phylogenetic diversity.

### Bovine nasopharyngeal microbiome beta diversity

The bacterial community structure (beta diversity) or distance between groups (healthy and treatment period) as determined by Bray–Curtis Dissimilarity and Weighted-UniFrac were not significantly different over time during the healthy or treatment periods (*p* > 0.05). Additionally, no significant difference was observed in the dispersion of the samples from the centroid of each day (*p* > 0.05). Interestingly, the bacterial community structure was significantly different as determined by Bray–Curtis dissimilarity [*F*(1, 7) = 2.0978, *R*^2^ = 25.9, *p* = 0.04, Supplementary Figure S4A] and Weighted UniFrac [*F* (1, 7) = 2.20, *R*^2^ = 26.84, *p* = 0.04, Supplementary Figure S4B], between the animals that received florfenicol or tulathromycin 1 day after administration (day 1). No significant difference in dispersion of the samples was observed on days 5 and 10. Lastly, no sickness effect was observed in the bacterial structure or distance as well as dispersion between apparently healthy animals from day 7 and BRD-affected animals sampled on day 0 (day of disease diagnosis).

### Cattle nasopharyngeal microbiota taxonomical composition

**Figure 1:**
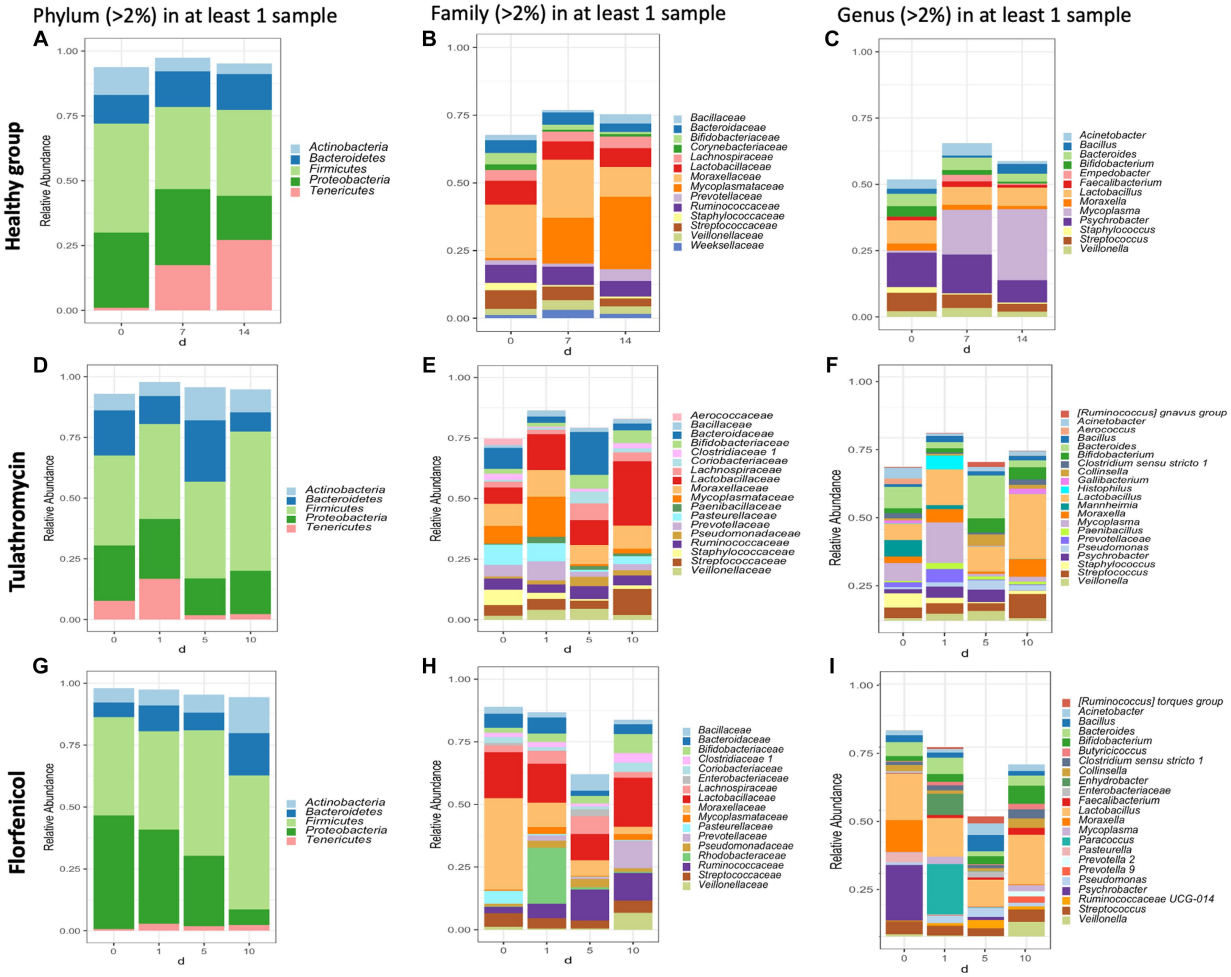
Taxonomic composition of phyla, families, and genera with an abundance >2% present in the nasopharynx of animals apparently healthy **(A–C)** and BRD-affected animals treated with tulathromycin **(D–F)** or florfenicol **(G–I)**. Nasopharyngeal samples of the apparently healthy animals were collected on days 0, 7 and 14 and NP of the BRD-affected animals were collected on the day of respiratory disease diagnosis (0 day), and 1, 5 and 10 days after antibiotic treatment administration.

**Figure 2:**
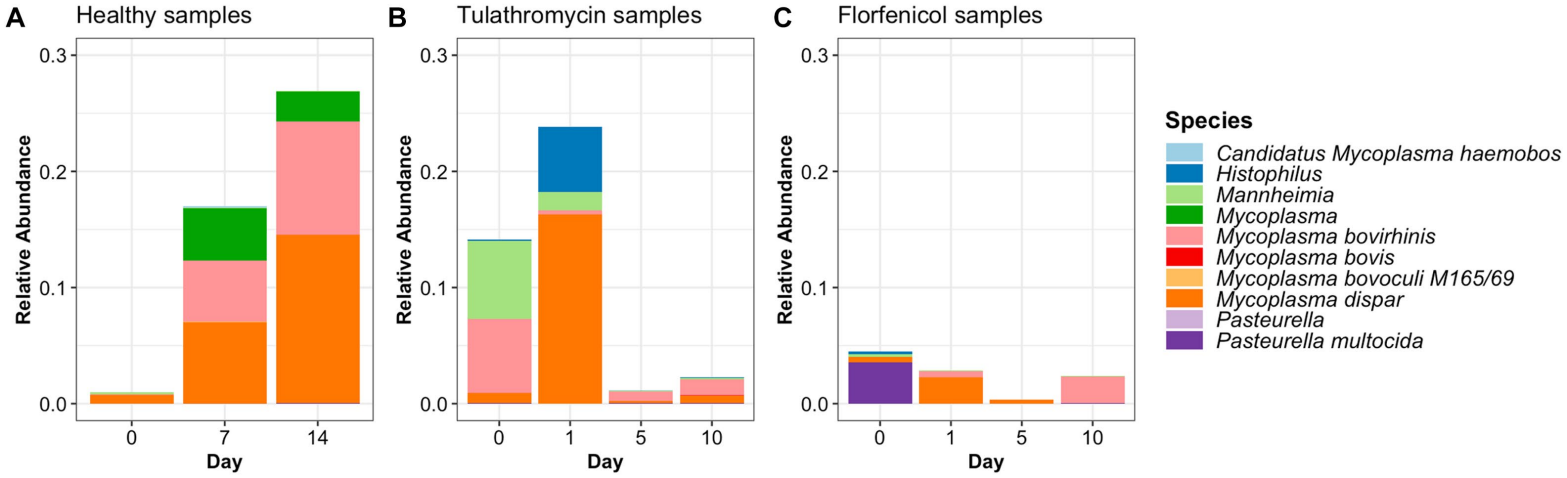
Relative abundance of BRD-pathobionts in the NP of apparently healthy calves **(A)** and BRD-affected calves treated with tulathromycin **(B)** or florfenicol **(C)**.

In this study, the top three phyla with the highest relative abundance in the NP microbiome across all timepoints in the animals from the healthy period were *Firmicutes* (35% of the community on average), *Proteobacteria* (25%), and *Tenericutes* (16%) (Figure 1A). At a family level, *Moraxellaceae* (17%), *Mycoplasmataceae* (17%), and *Lactobacillaceae* (7%) were the most abundant (Figure 1B) and at a genus level, *Mycoplasma* (16%), *Psychrobacter* (12%), and *Lactobacillus* (7%) were the most abundant (Figure 1C). *Mycoplasma* species, specifically *M. dispar* (8.2%) and *M. bovirhinis* (5.6%) were the most abundant *Mycoplasma* ASVs in the healthy animals (Supplementary Figure S5A). Additionally, it appeared that *Psychrobacter* and *Mycoplasma* are in inversely associated as time passed. More specifically, *Mycoplasma* and *Pyschrobacter* had a relative abundance of 0.8% and 12.9%, respectively, on day 0, while on day 14, most of the community was composed of *Mycoplasma* (26.8%) and less of *Psychrobacter* (8.34%). ASVs identified as *Histophilus* and *Mannheimia*, and the species *P. multocida* and *M. bovis* were found in the apparently healthy samples; nevertheless, these genera and species had a relative abundance <1% across all the different timepoints (Figure 2A).

In the case of the animals treated with tulathromycin or florfenicol, the NP community regardless of the antibiotic treatment was composed of the phyla *Firmicutes*, *Proteobacteria* and *Bacteroides* (Figures 1D,G), the families *Lactobacillaceae* and *Enterobacteriaceae* (Figures 1E,H) and the genera *Lactobacillus* followed by *Escherichia-Shigella* (Figures 1F,I). Interestingly, on day 0, when clinical signs were observed, the most abundant genera in the animals treated with tulathromycin were *Bacteroides* (8.71%), *Mycoplasma* (7.25%), and *Mannheimia* (6.74%) (Figure 1F). On the other hand, the NP composition in the animals before being treated with florfenicol, day 0, was mostly composed by *Psychrobacter* (21.92%), *Lactobacillus* (18.35%), and *Moraxella* (12.26%) (Figure 1I).

After the animals received tulathromycin or florfenicol on day 1, the NP microbiome in the two antibiotic groups had similarities and differences. In the samples treated with tulathromycin, *M. dispar* increased from day 0 to 1% to 16.6%, while *M. bovirhinus* decreased from day 0 to day 1. Both these species decreased dramatically 5 and 10 days post-tulathromycin administration (Supplementary Figures S5B,C). We then identified the sequences percent identify between *M. dispar*, *M. bovirhinis* compared to *M. bovis*, a known bacterium related to cause respiratory disease in cattle. Interestingly, ASVs assigned to *M. bovirhinis* and *M. dispar* were on average 91.08% and 83.25% identical, respectively, to the ASV identified as *M. bovis* (Supplementary Table S1). Additionally, in the samples treated with tulathromycin, the genera *Mannheimia* decreased to 1.6% on day 1 and remained with an abundance <1% as time passed and the genera *Histophilus* increased from less than 2% in a sample on day 0 to 5.59% on day 1 but decreased through time (Figure 1F). In the florfenicol group, *Paracoccus* increased from an abundance less than 2% on day 0 to 19.91% on day 1 and decreased dramatically 5 and 10 days after florfenicol administration. Additionally, the abundance of *Psychrobacter* sp., decreased from 21.92% to 0.15% on day 1. Additionally, the presence of *Pasteurella* was abundant on day 0 (3.56%) but decreased at time passed (<1%) and *Mycoplasma* abundance was high 1 day and (day 1, 2.79%) and 10 days after florfenicol treatment (2.29%) (Figure 1I).

One similarity in the NP composition between the samples treated with tulathromycin or florfenicol is that *Lactobacillus* was abundant at each time point (Figure 1). In particular, the abundance of NP microbial communities between both antibiotic groups on day 10 were dominated by *Lactobacillus*, followed by *Streptococcus* and *Bifidobacterium*. A similar result was observed in the healthy samples, in which *Lactobacillus* is abundant across all timepoints. We then sought to identify the relative abundance of the *Lactobacillus* species in the healthy and treatment periods (Supplementary Figure S6). Unfortunately, the taxonomic classifier utilized in this study failed to identify the species taxonomy of 117 ASVs identified as *Lactobacillus* sp. which composed approximately 1% and 15% of the NP microbiome relative abundance in the healthy and treatment period, respectively. To determine the species composition from the 117 *Lactobacillus* ASV, we utilized Greengenes 13_8, 515/806 region database and BLAST (Supplementary Figure S6). After assigning the new taxonomy, the *Lactobacillus* species *L. salivarius*, *L. helveticus*, *L. mucosae*, *L. gasseri*, and *L. amylovorus* were the most abundant regardless of disease status. Interestingly, the abundance of *L. gasseri* increased after tulathromycin treatment and decreased in the animals treated with florfenicol whereas *L. salivarius* increased in relative abundance as time passed regardless of antibiotic treatment (Supplementary Figure S6). Lastly, a similar numerical inverse association between *Psychrobacter* and *Mycoplasma* was observed in both BRD-affected groups.

#### Change in the BRD-pathobionts relative abundance before and after antibiotic treatment

ASVs assigned to the BRD pathobionts *Histophilus* and *Mannheimia* and the species *M. bovis* and *P. multocida* were found on the day of disease identification (day 0). From among those BRD pathobionts, only the *Mannheimia* genus, in the tulathromycin group (Figure 2B), and *P. multocida*, in the florfenicol group (Figure 2C), had a relative abundance greater than 1% on day 0, but their abundance decreased after antibiotic administration (<1%). *Histophilus* sp. and *M. bovis* were detected on day 0 with a relative abundance of <1% and remained with low abundance after antibiotic treatment. Interestingly, the abundance of the BRD-pathobionts during the treatment period was dependent on the calf (Tables 1, 2). In the group treated with tulathromycin (Table 1), calf 29 had a higher relative abundance of *Histophilus* (0.46%) on day 0 and decreased as time passed. Additionally, calf 21 was the only animal with a high relative abundance of *Mannheimia* on day 0 (33.21%) but decreased as time passed while calf 28 had a relative abundance of 0.18% on day 0 but increased 5 days after tulathromycin treatment (0.29%). A different pattern was observed for calf 9 from which *Histophilus* was abundant after tulathromycin administration, day 1. *M. bovis* had low relative abundance except for calf 18 on day 10 after tulathromycin treatment (0.22%). In the case of the florfenicol group (Table 2), *Mannheimia* was abundant in calf 19 on day 0 but abundance changed as time passed and calf 30 had a higher abundance of *Histophilus*, *Mannheimia*, and *P. multocida* on the day of disease diagnosis. No BRD-pathobiont was observed on calf 15 except until day 10 from which *P. multocida* was abundant. Again, no *M. bovis* abundance was observed on the calves treated with florfenicol. Additionally, we then identified the relative abundance of the BRD-pathobionts in the NP of paired healthy animals not treated with antibiotics (Table 3). Interestingly, the relative abundance of the BRD-pathobionts in these animals was <1% and variable across all the animals.

**Table 1:** Relative abundance of ASVs classified as BRD-pathobionts in the NP of the BRD-affected animals treated with tulathromycin.

Calf	*Histophilus*				*Mannheimia*				*P. multocida*				*M. bovis*			
	Day 0	Day 1	Day 5	Day 10	Day 0	Day 1	Day 5	Day 10	Day 0	Day 1	Day 5	Day 10	Day 0	Day 1	Day 5	Day 10
9	0	33.6	NA	0	0	0	NA	0	0	0	NA	0.25	0	0	NA	0
18	0	0	0	0	0.52	9.45	0	0	0	0	0	0	0	0	0	0.2
21	0	0	0.11	0.32	33.2	0	0	0	0	0	0	0	0	0	0	0
23	NA	0	0	0	NA	0	0	0.59	NA	0	0	0	NA	0	0	0
28	0	0	0	0	0	0	0	0	0.18	0	0.29	0	0	0	0	0
29	0.46	0	0	0	0	0.16	0.12	0.11	0	0	0	0	0	0	0	0

NA, sample not included due to low sequence counts (<1,277 reads).

**Table 2:** Relative abundance of ASVs classified as BRD-pathobionts in the NP of the BRD-affected animals treated with florfenicol.

Calf	*Histophilus*				*Mannheimia*				*P. multocida*				*M. bovis*			
	Day 0	Day 1	Day 5	Day 10	Day 0	Day 1	Day 5	Day 10	Day 0	Day 1	Day 5	Day 10	Day 0	Day 1	Day 5	Day 10
15	NA	0	0	0	NA	0	0	0	NA	0	0	0.12	NA	0	0	0
19	0	0	0	0	0.22	0.24	0	0.05	0	0	0	0	0	0	0	0
30	0.44	0	NA	0	0.22	0	NA	0	7.13	0	NA	0.05	0	0	NA	0

NA, sample not included due to low sequence counts (<1,277 reads).

**Table 3:** Relative abundance of ASVs classified as BRD-pathobionts in the NP of the healthy animals not treated with antibiotics (*n* = 11).

Calf	*Histophilus*			*Mannheimia*			*P. multocida*			*M. bovis*		
	Day 0	Day 7	Day 14	Day 0	Day 7	Day 14	Day 0	Day 7	Day 14	Day 0	Day 7	Day 14
2	0	0	0	0	0	0	0	0	0	0	0	0
4	0	0	0	0.17	0	0.73	0	0	0	0	0	0.07
6	0	0	0	0	0	0	0	0	0.99	0	0	0
8	0	0	0	0	0	0	0	0	0	0	0	0
11	0	0	0	0.06	0.12	0	0	0.24	0	0	0	0
12	0	0	0	0	0	0	0	0	0	0	0	0
13	0	0	0	0	0.59	0	0	0	0	0	0	0
17	NA	0	0	NA	0	0.37	NA	0	0	NA	0	0
20	0	0	0	0.34	0	0	0	0	0	0	0	0
22	0	0	0	0	0	0.16	0	0	0	0	0	0
25	NA	0	0	NA	0	0.05	NA	0	0	NA	0	0

NA, sample not included due to low sequence counts (<1,277 reads).

### Beta regression

**Figure 3:**
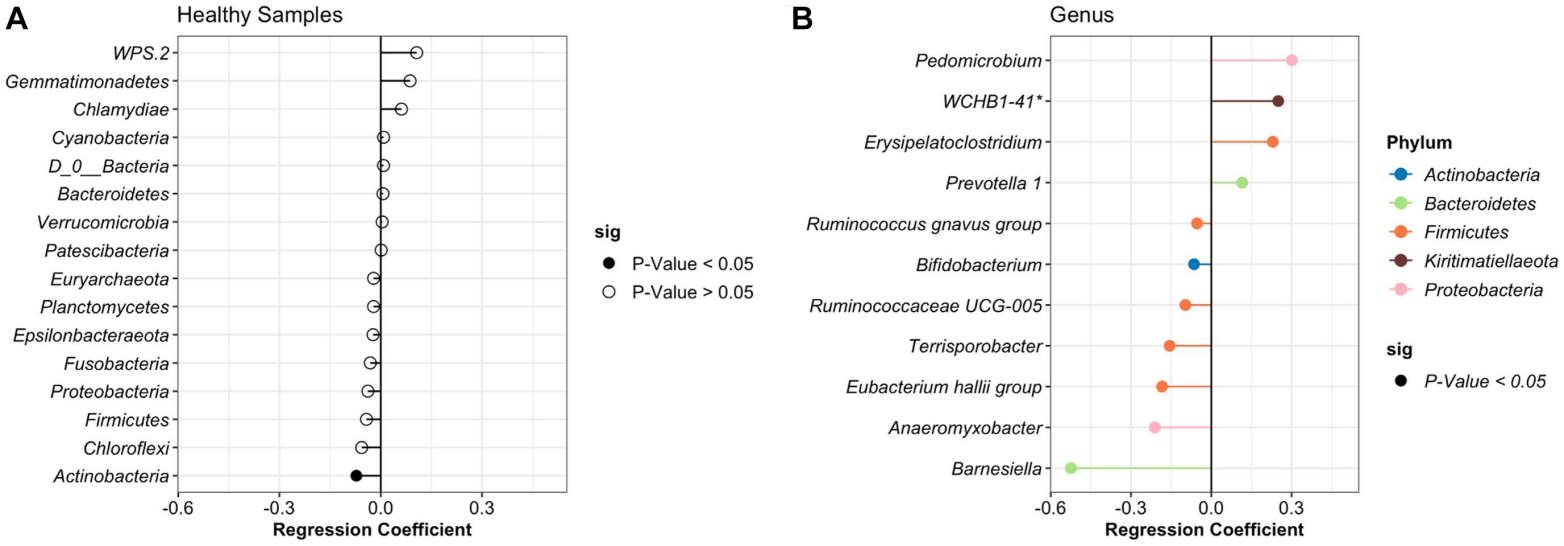
Differentially abundant phylum **(A)** and genus **(B)** in the healthy animals as time passed. Regression coefficient calculated using a beta regression, determine the change in abundance: increase >0 and decrease <0.

After identifying what are the most abundant taxa for each period (healthy or treatment) and day, we then sought to identify specific taxa that changed over time using beta regression. For the analysis, we applied a filter to only include phyla with an average abundance greater than >0.0001 and genera with an average abundance greater than >0.001. A total of 37 different phyla and 916 genera were identified in the study; however, after applying the filters, 27 phyla and 110 genera remained and were used for the analysis. For the healthy samples, the phyla *Actinobacteria* significantly decreased over time. At the genus level, 5 groups increased, and 7 groups significantly decreased over time (Figure 3). However, these bacteria had a relative abundance of <2% in the NP community composition. Additionally, no significant difference was observed in the relative abundance of the BRD-pathobionts identified in the NP of the apparently healthy group as time passed.

**Figure 4:**
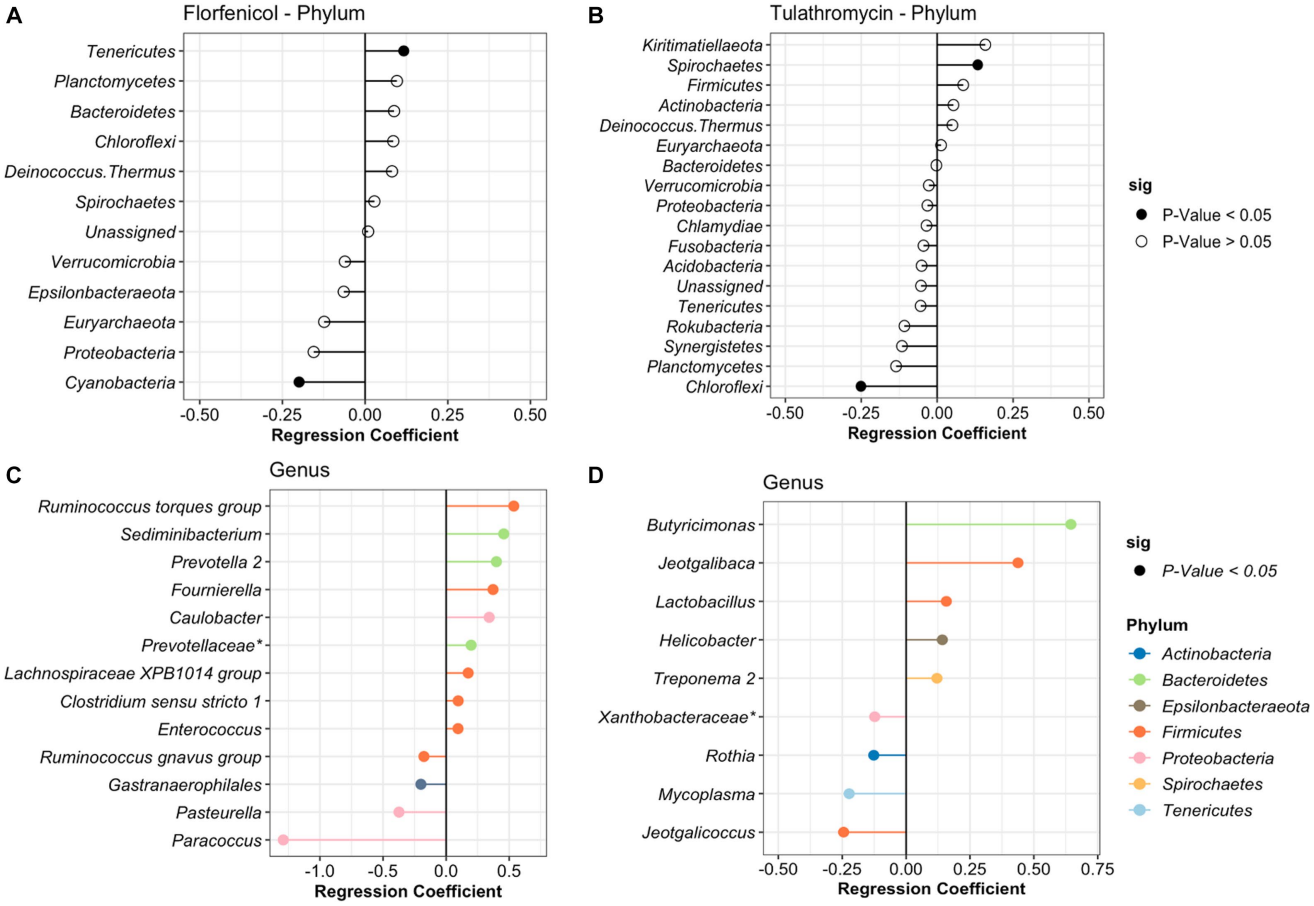
Differentially abundant phyla and genera in the nasopharynx of animals diagnosed with respiratory disease treated with florfenicol **(A,C)** or tulathromycin **(B,D)**. Regression coefficient calculated using a beta regression, determine the change in abundance: increase >0 and decrease <0.

In the case of the treatment period, samples were divided based on the antibiotic treatment (tulathromycin and florfenicol). For calves that received florfenicol, bacteria from the phylum *Tenericutes* significantly increased while *Actinobacteria* decreased with time. Whereas in the tulathromycin group, *Spirochaetes* increased and *Cholofexi* decreased (Figure 4). At genus level in the florfenicol group, 9 genera increased and 4 decreased including *Pasteurella*. Again, these differentially abundant bacteria had a relative abundance of <2%. However, beta regression modeling at the species level did not reveal any significant pattern or change in the relative abundance of specific *Pasteurella* species. Only three bacterial species, *Veillonella* sp. (estimate: 0.359)*, Paracoccus* sp. (estimate: −1.217), and *Psychrobacter* sp. (estimate: −0.819) had significant changes in their abundance as time passed (*p* < 0.05). On the other hand, fewer genera groups increased in the tulathromycin group as time passed for a total of 5, including the genera *Lactobacillus* and 4 groups decreased, from which one of them was the genera *Mycoplasma* (Figure 4). However, beta regression modeling at the species level did not reveal any significant pattern or change in the relative abundance of specific *Mycoplasma* species in the tulathromycin group as time passed and only *Lactobacillus* sp. increased as time passed (regression estimate: 0.126, *p* = 0.014). No other significant difference was observed in the florfenicol group.

**Figure 5:**
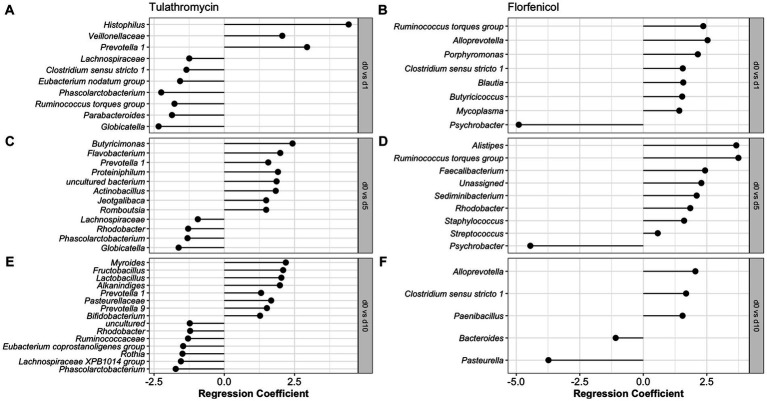
Comparison of the NP microbiome between day of diagnosis (day 0) and after tulathromycin **(A,C,E)** or florfenicol **(B,D,F)** treatment (days 1, 5 and 10). Regression coefficient calculated using a beta regression, determine the change in abundance: increase >0 and decrease <0 compared to day 0.

To identify the antibiotic effect and recovery on the NP of the BRD-affected group, a beta regression was performed for the calves treated with tulathromycin (*n* = 6) and florfenicol (*n* = 3). For this analysis, NP samples collected on day 1 (after antibiotic treatment), days 5 and 10 were compared to day 0 (day of disease diagnosis). The antibiotic effect was identified by comparing days 0 and 1 in the tulathromycin and florfenicol groups (Figures 5A,B). Nasopharyngeal composition on the tulathromycin group between days 0 and 1 revealed that the relative abundance of *Histophilus* significantly increased after antibiotic administration (day 1) similar to the relative abundance of unclassified *Veillonellaceae* and *Prevotella* 1 and 7 NP members decreased after tulathromycin administration (Figure 5A). On the other hand, more NP members (*n* = 7) increased after florfenicol administration compared to the tulathromycin effect. From these members, *the Ruminococcus torques* group, *Alloprevotella*, and *Porphyromonas* had higher relative abundance on day 1 compared to day 0. Additionally, *Mycoplasma* relative abundance increased after florfenicol treatment while the *Psychrobacter* decreased (Figure 5B).

The nasopharyngeal microbiome recovery from antibiotic treatment was different for each of the groups. Again, the relative abundance of *Prevotella* 1 was higher 5 days after the tulathromycin group compared to day 0. Other members including *Butyricimonas* and *Flavobacterium* had higher relative abundance on day 5 and only 4 NP members had lowered relative abundance when compared to day 0 (Figure 5C). Additionally, the genera *Phascolarctobacterium* was decreased on all days compared to day 0. In the florfenicol group, 5 days after the calves were treated, the relative abundance of *Alistipes*, *Ruminoccocus torques* group was higher compared to day 0. Interestingly, ASVs classified as *Staphylococcus* and *Streptococcus* were significantly higher on day 5 compared to day 0. Again, the relative abundance of *Psychrobacter* was lower on day 5 compared to day 0 (Figure 5D). Lastly, 10 days after receiving tulathromycin, the NP microbiome of these calves had a higher abundance of *Myroides*, *Fructobacillus*, *Lactobacillus*, *Alkanindiges*, and again, *Prevotella* 1 compared to day 0 (Figure 5E). However, 10 days after animals were treated with florfenicol a higher abundance of *Alloprevotella*, *Clostridium sensu stricto* 1, and *Paenibacillus* while a lower abundance of *Bacteroides* and *Pasteurella* was observed compared to day 0 (Figure 5F).

DESeq analysis was used to identify differentially abundant taxa between the healthy day 7 samples and sick day 0 samples. Only one bacterium, *Blautia* sp., was significantly higher in the healthy animals than the sick animals (log_2_ fold change = 3.81, *p* = 0.01). No other differentially abundant bacteria were observed between the two groups.

## Discussion

In this study, we described the ecology of the NP microbiota in pre-weaned and apparently healthy or BRD-affected Holstein calves after transport. Our study indicates that specific members of the NP microbiome changed over time or as the animal aged. On the contrary, the NP microbiome, specifically the richness and phylogenetic diversity, remains constant with no significant changes due to time or disease development. Contrary to our initial hypothesis, intramuscular and subcutaneous antibiotic administration did not decrease the NP alpha diversity in the BRD-affected animals. However, the NP bacterial community structure of the BRD-affected animals after antibiotic administration was different between the florfenicol and tulathromycin groups. We observed that florfenicol administration seemed to increase the relative abundance of more NP members than tulathromycin administration, with 7 members increased after florfenicol compared to the 3 members increased in the tulathromycin group. In a previous study testing the effect of a single injection of tulathromycin and oxytetracycline in the feedlot NP, the authors observed that the NP of both antibiotic treatments was affected during the first 5 days after treatment administration and their microbiota communities remained dissimilar across the study (31). Thus, this confirms that the antimicrobial treatment selected and/or its route of administration (IM vs. subcutaneous) have an effect on the NP microbiome community and remain different even 10 days after treatment.

Findings in this study demonstrated that the NP microbiome in the healthy animals during the first 2 weeks after arrival was mostly affected by time, with specific members changing and with no direct association with health status. Nonetheless, at a genus level, the healthy animals presented a high relative abundance of *Mycoplasma* (mostly *M. dispar* and *M. bovirhinis*), *Escherichia-Shigella*, and *Psychrobacter*. In our study, we observed the presence of the genera *Histophilus* and *Mannheimia*, as well as the species *M. bovis* and *P. multocida* in both the apparently healthy and BRD-affected animals. In the case of the apparently healthy group, the abundance of these BRD-pathobionts was lower than 1% of the NP community. On the contrary, the genera *Mannheimia* found in the tulathromycin group and the species *P. multocida* found in the florfenicol group, composed approximately 3% and 7% of the NP community on the day of diagnosis, respectively. Nonetheless, after comparing the abundance of these pathogens present in BRD-affected animals compared to the apparently healthy, no significant difference was observed in the relative abundance of these pathobionts between the two groups, contrary with our initial hypothesis. Additionally, it was observed that abundance of these pathobionts in the NP was dependent on the calf and not associated with health status.

Even though we observed *Mycoplasma* species, such as *M. dispar* and *M. bovirhinis*, had a high relative abundance on days 0 and 1 in the tulathromycin group, beta regression modeling did not reveal any significant change in their abundance over time. These two *Mycoplasma* species have been previously identified as opportunistic pathogens related to bovine respiratory disease (32, 33). More specifically, *M. dispar* has been detected in pneumonic cases in cattle and can exhibit a pathogenic phenotype by suppressing the host immune response, and producing hydrogen peroxide and biofilms (34). Nonetheless, another study identified a high relative abundance of *M. dispar* in the nasopharynx of apparently healthy heifers after feedlot arrival (35) similar to the results observed in our study. Indeed, the presence of *M. dispar* tends to be linked with respiratory disease development in cattle; however, in our study, this bacterium was not differentially abundant between apparently healthy animals compared with those that developed signs of BRD. In addition, high abundance of *M. dispar* was observed only in animals treated with tulathromycin and not in the florfenicol group. This may be due to the small number of animals in both these groups. Other studies had proposed that *M. dispar* acts as an indicator of good respiratory health or provides the host with some level of resilience against *M. bovis* (12, 35). Unfortunately, we do not possess enough information to determine if the presence of *M. dispar* in our study is an indicator of respiratory health based on the high relative abundance observed in the apparently healthy and BRD-affected animals. In our study, it was observed that the genus *Psychrobacter* had high relative abundance in the NP of apparently healthy animals as well as in the NP BRD-affected animals before receiving florfenicol and remain apparently abundant in animals receiving tulathromycin. In addition, it appeared that *Psychrobacter* was numerically inverse associated with *Mycoplasma* regardless of disease diagnosis and antibiotic treatment. Studies had identified *Psychrobacter* as a normal commensal of the upper respiratory tract in dairy cattle (12, 17). Others have demonstrated that *Psychrobacter* in the NP of healthy feedlot cattle is potentially inversely associated with *Mycoplasma* (17). A numerical inverse relationship was observed between *Psychrobacter* and *Mycoplasma* in our study (not statistically significant), potentially highlighting an antagonistic interaction between these two genera. Nonetheless, more *in silico* and *in vitro* research is needed to determine the interactions between these two bacterial species.

Lastly, we observed in this study that the abundance of *Lactobacillus* sp. was similar between the apparently healthy and BRD-affected animals; however, the abundance of this genera increased 10 days after regardless of antibiotic treatment and could potentially indicate recovery from respiratory disease. At a species level, it was observed that *L. gasseri*, *L. helviticus*, *L. mucosae*, *L. salivarius*, *L. amylovorus* were common members of the NP in both apparently healthy and BRD-affected animals. From these species, the abundance of *L. gasseri* increased as time passed in the tulathromycin group while it decreased on the florfenicol group. On the other hand, *L. salivarius* increased as time passed regardless of antibiotic treatment. Additionally, *L. amylovorus* had low relative abundance on the apparently healthy animals whereas its abundance increased in the BRD-affected animals after tulathromycin and florfenicol administration, potentially indicate recovery after antibiotic administration. These *Lactobacillus* strains have been identified as potential probiotics in humans due to their proteolytic capacities, and different antagonistic activity such as occupy the niche of pathogens present in the gastrointestinal tract and bacteriocins production against other members in the community (36–41). Regardless of its known probiotic capacities in humans, more research is needed to determine if these *Lactobacillus* species can act as probiotics to potentially inhibit or decrease respiratory disease incidence in cattle. Only few *Lactobacillus* strains, specifically, *L. amylovorus*, have been previously found to inhibit *M. haemolytica* growth through competition and adherence to bovine turbinate cells *in vitro* and *in vivo* (42, 43); nonetheless, it is necessary to determine its probiotic capacity against other BRD-pathobionts. Based on these results, the taxonomic composition patterns shared between the apparently healthy and BRD-affected animals can be explained by the hypothesis that respiratory development in these groups of animals is not solely associated with the presence of BRD-pathobionts in the NP. Thus, more research is needed to account not only for the NP composition to potentially explain disease etiology but include the presence of other factors such as a change in environmental temperature, and the presence of viruses and fungi related to respiratory development. Lastly, the numerical increase of *Lactobacillus* after antibiotic administration and the presence of known human probiotics in the calf NP microbiome could be further investigated to determine if the presence of these *Lactobacillus* strains is an indicator of antibiotic recovery and potential probiotic use to prevent respiratory disease in cattle.

## Conclusion

The specific members of NP microbiome of pre-weaned and post-transport calves that remained healthy throughout the study appears to be affected by age. The alpha diversity of the BRD-affected group was not affected by either tulathromycin or florfenicol administration when compared to apparently healthy calves. Nonetheless, differences in the NP microbiome composition, microbiome relative abundance, and beta diversity were observed in tulathromycin treated compared to florfenicol treated calves. Specific members of the NP microbiome were affected 1 day after tulathromycin or florfenicol administration and the calves microbiome remained different 10 days after treatment. Our data suggest that BRD development was not solely determined by the abundance of BRD-associated bacteria (*P. multocida*, *H. somni*, *M. bovis*, and *M. haemolytica*) in the NP microbial community. Additionally, antibiotic administration did not decrease the abundance of BRD-pathobionts and differences in BRD-pathobiont relative abundance was dependent on the calf. Additionally, 10 days after antibiotic treatment, there was a numerical increase of *Lactobacillus* strains in the NP of antibiotic-treated cattle, indicating the potential of these species to act as probiotics to prevent respiratory disease. Lastly, more research is needed to understand how BRD develops through time and if other bacteria, fungi, viruses, or their interactions play a role in disease initiation and development.

## Data availability statement

The original contributions presented in the study are publicly available. This data can be found at: https://github.com/EuniceCenteno/calf.nasopharyngeal; Nasopharyngeal sequences were deposited in the NCBI sequence read archive (SRA) database under Bioproject: PRJNA1015679, BioSamples: SAMN37361853- SAMN37361960.

## Ethics statement

The animal study was approved by Purdue University Institutional Animal Care and Use Committee (Protocol #1808001783). The study was conducted in accordance with the local legislation and institutional requirements.

## Author contributions

RC-M: Formal analysis, Investigation, Visualization, Writing – original draft, Writing – review & editing. RK: Conceptualization, Investigation, Writing – review & editing. JK: Conceptualization, Investigation, Writing – review & editing. JB: Conceptualization, Investigation, Methodology, Writing – review & editing. TJ: Conceptualization, Funding acquisition, Investigation, Methodology, Project administration, Supervision, Writing – review & editing.
