# Supplementary material for: Dynamics of the nasopharyngeal microbiome of apparently healthy calves and those with clinical symptoms of bovine respiratory disease from disease diagnosis to recovery
Source: Front Vet Sci. 2023 Nov 16;10:1297158. doi: 10.3389/fvets.2023.1297158 (PMC10687565; doi:10.3389/fvets.2023.1297158)
Supplement: Supplementary file 1 [file Data_Sheet_1.docx]

**SUPPLEMENTARY MATERIAL**

**Dynamics of the nasopharyngeal microbiome of apparently healthy and calves with clinical symptoms of BRD from disease diagnosis to recovery.**

**Ruth Eunice Centeno-Martinez, Rebecca N. Klopp, Jennifer Koziol, Jacquelyn P. Boerman, Timothy A. Johnson.**

**Figure S1.** Relative abundance of the ASVs contaminants in the apparently healthy **(A, B, C),** BRD-affected animals treated with Tulathromycin **(D, E, F),** and Florfenicol **(G, H, I).**

**Figure S2.** Relative abundance of potential contaminants in the negative and positive controls. Colors represent different ASVs identified as *Escherichia-Shigella* **(A),** *Lactobacillus* **(B),** and *Clostridium sensu stricto* 1 **(C).** Samples labelled as “C_kitneg” are the negative control samples (only PCR grade water was added to DNA extraction tubes).”

**Figure S3.** Milk replacer intake averaged for the first two weeks of life between the BRD-affected (BRD) and apparently healthy animals (healthy).

**Figure S4.** Holstein calves’ nasopharyngeal bacterial community structure determined by Bray-Curtis dissimilarity **(A)** and Weighted UniFrac **(B)** based on antibiotic treatment.

**Figure S5.** Relative abundance of *Mycoplasma* in the NP of healthy animals **(A)** and BRD-affected animals treated with Tulathromycin **(B)** and with Florfenicol **(C**).

**Figure S6.** Relative abundance of *Lactobacillus* in the NP of healthy animals **(A)** and BRD-affected animals treated with Tulathromycin **(B)** and with Florfenicol **(C**).

**Supplementary Table 1.** Relative abundance of the significant differently abundant taxa over time in the apparently healthy animals and BRD-affected animals treated with Tulathromycin or Florfenicol.

**Supplementary Table 2.** Information of the BRD-affected animals NP sample collection**.**


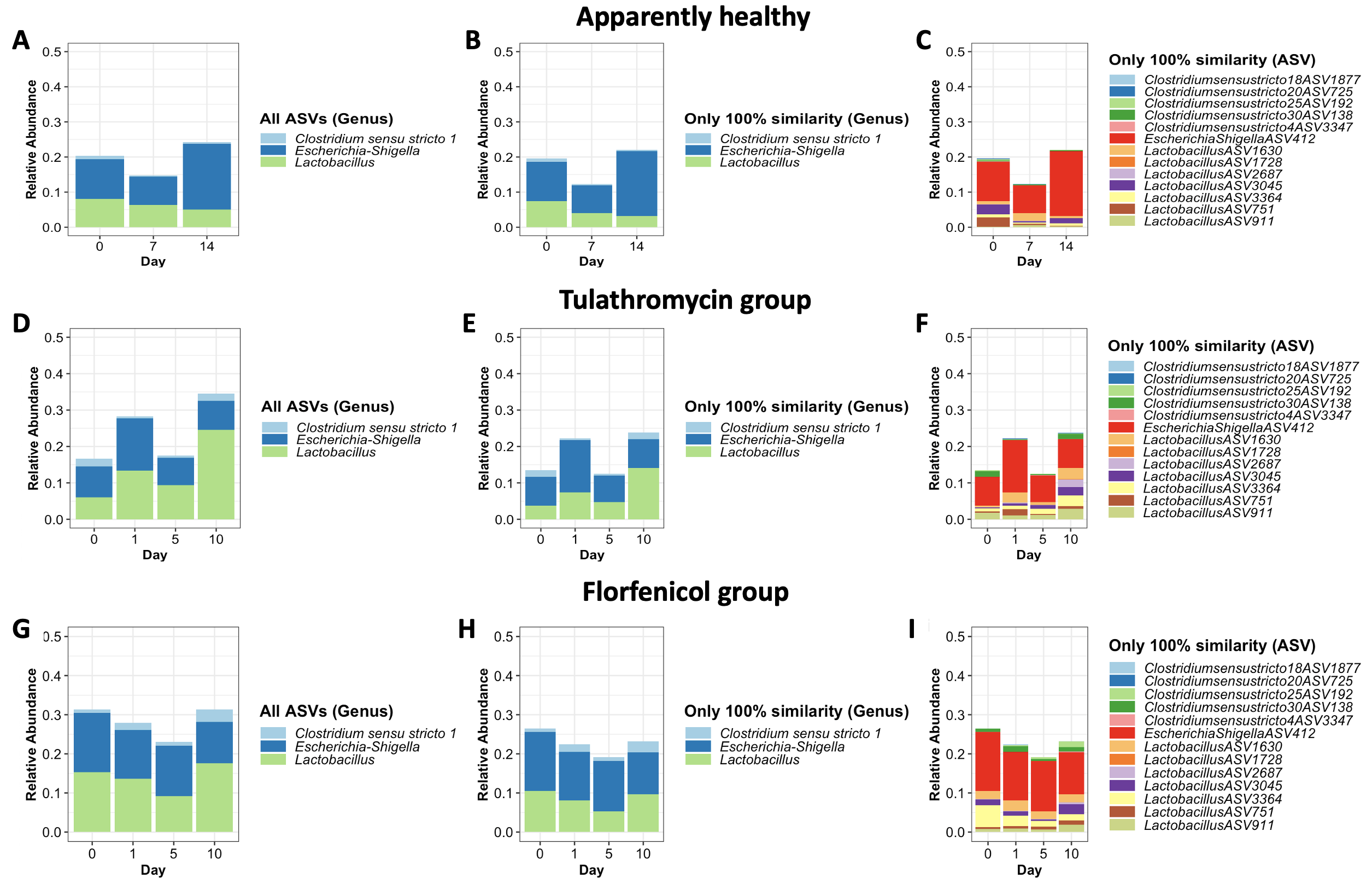


**Figure S1.** Relative abundance of the ASVs contaminants in the apparently healthy **(A, B, C),** BRD-affected animals treated with Tulathromycin **(D, E, F),** and Florfenicol **(G, H, I).**


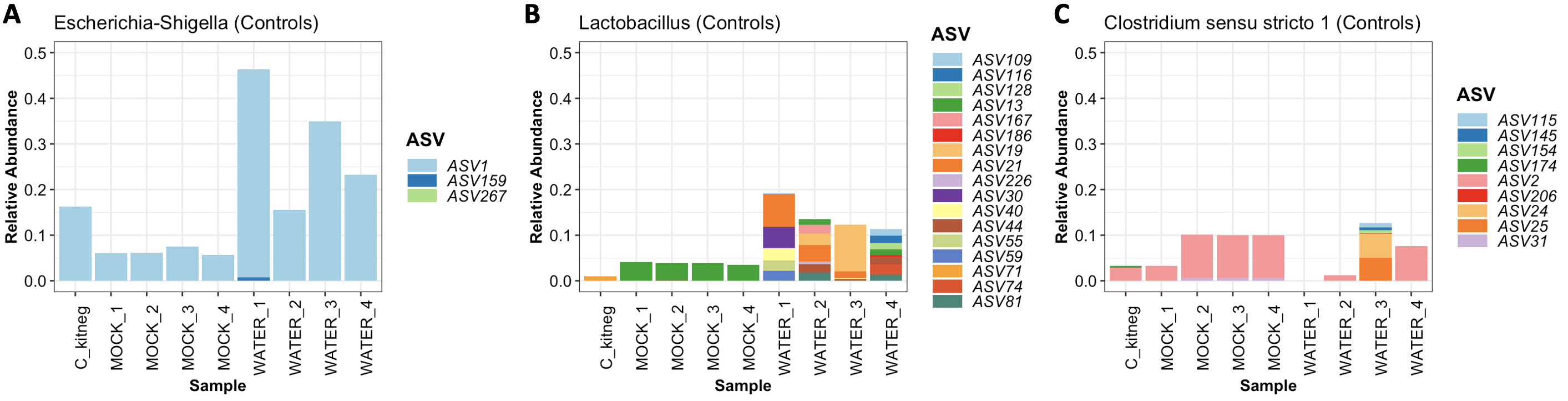


**Figure S2.** Relative abundance of potential contaminants in the negative and positive controls. Colors represent different ASVs identified as *Escherichia-Shigella* **(A),** *Lactobacillus* **(B),** and *Clostridium sensu stricto* 1 **(C).** Sample labelled as C_kitneg represents the negative control used during DNA extraction.

**
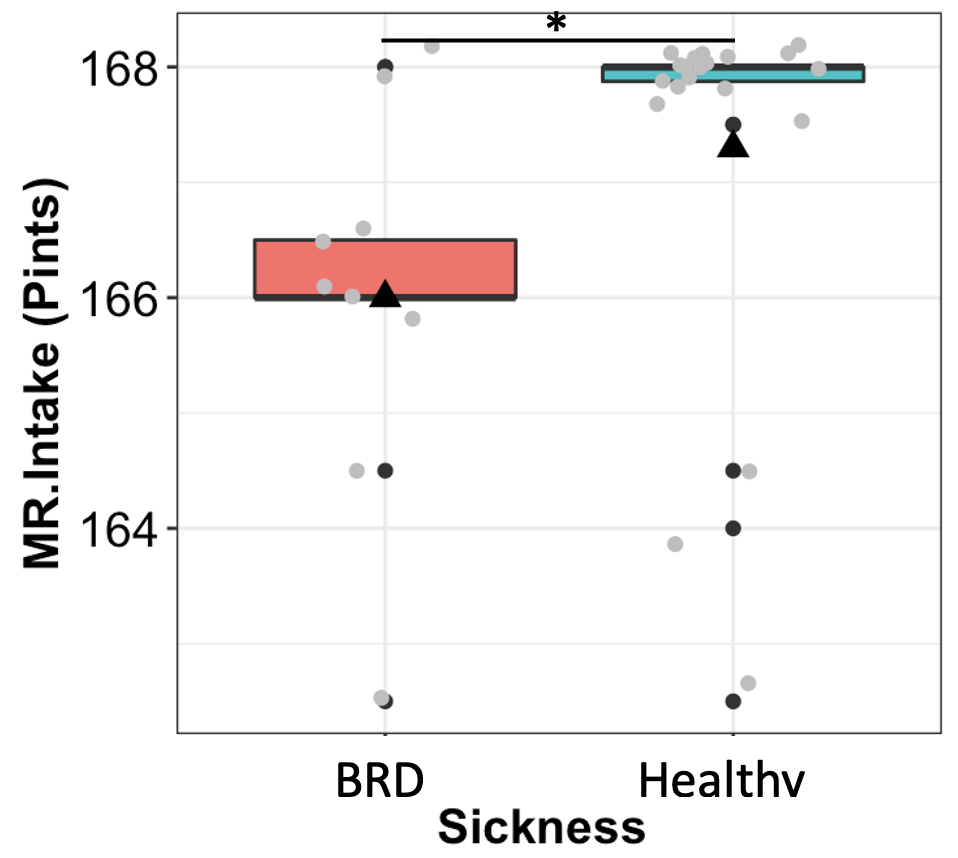
**

**Figure S3.** Milk replacer intake averaged for the first two weeks of life between the BRD-affected (BRD) and apparently healthy animals (healthy).


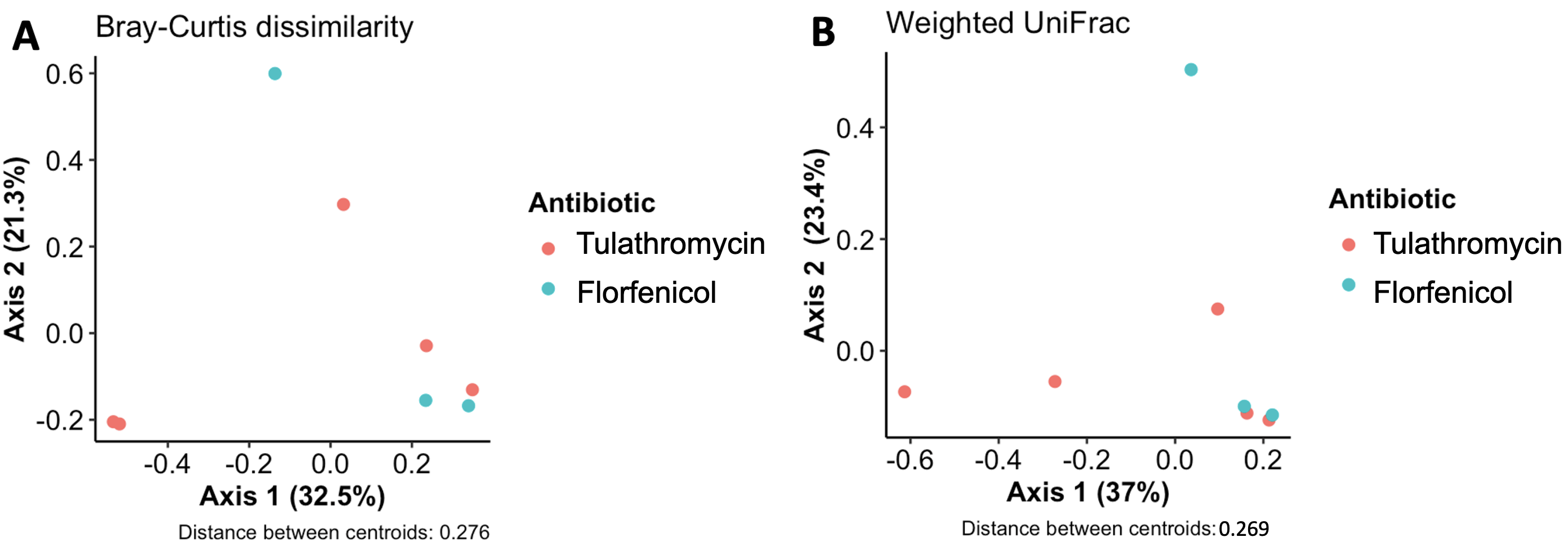


**Figure S4.** Holstein calves’ nasopharyngeal bacterial community structure determined by Bray-Curtis dissimilarity **(A)** and Weighted UniFrac **(B)** one day after antibiotic treatment.

**
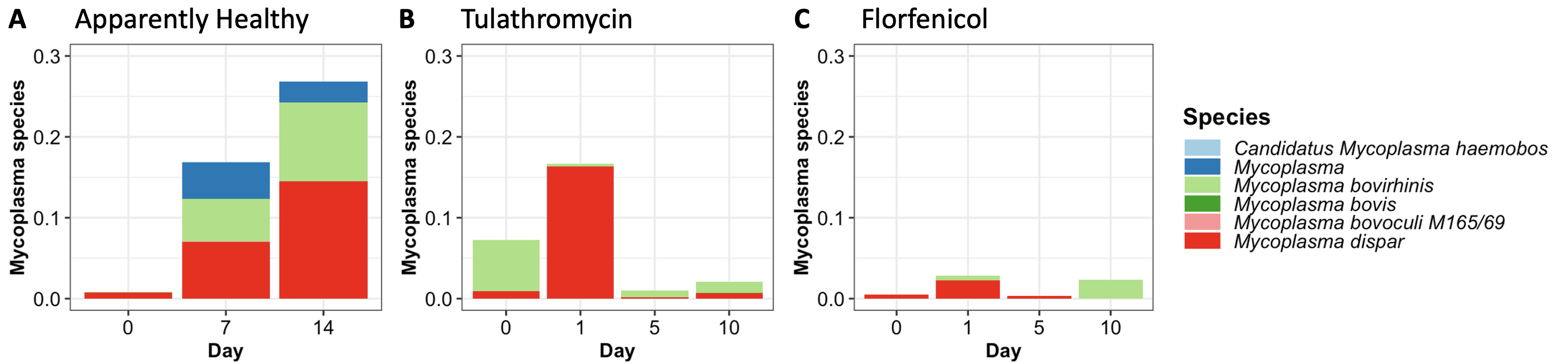
**

**Figure S5.** Relative abundance of *Mycoplasma* in the NP of healthy animals **(A)** and BRD-affected animals treated with Tulathromycin **(B)** and with Florfenicol **(C**).


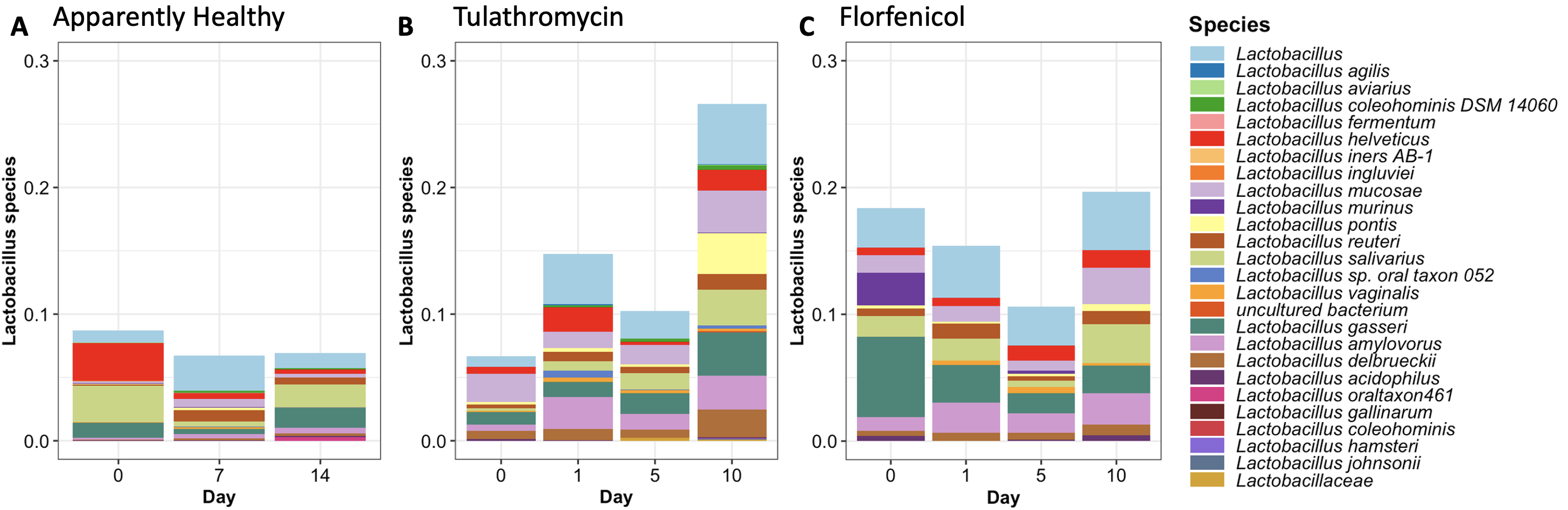


**Figure S6.** Relative abundance of *Lactobacillus* in the NP of healthy animals **(A)** and BRD-affected animals treated with Tulathromycin **(B)** and with Florfenicol **(C**).

| **Supplementary Table 1.** Relative abundance of the significant differentially abundant taxa over time in the apparently healthy animals and BRD-affected animals treated with Tulathromycin or Florfenicol. | | | | | | | | |
| --- | --- | --- | --- | --- | --- | --- | --- | --- |
| **Group** | **ASV** | **estimate** | **std.error** | **statistic** | ***P*** | ***P.adj*** | **Genus** | **Rel Abun%** |
| Apparently Healthy | ASV40 | -0.065 | 0.023 | -2.808 | 0.0050 | 0.0050 | *Bifidobacterium* | 1.9879 |
|  | ASV483 | -0.054 | 0.026 | -2.038 | 0.0416 | 0.0416 | *Ruminococcus gnavus* group | 0.2578 |
|  | ASV525 | -0.097 | 0.042 | -2.282 | 0.0225 | 0.0225 | *Ruminococcaceae* UCG-005 | 0.2357 |
|  | ASV191 | 0.115 | 0.056 | 2.043 | 0.0410 | 0.0410 | *Prevotella 1* | 0.2343 |
|  | ASV168 | -0.525 | 0.105 | -4.981 | 0.0000 | 0.0000 | *Barnesiella* | 0.2263 |
|  | ASV654 | 0.301 | 0.063 | 4.738 | 0.0000 | 0.0000 | *Pedomicrobium* | 0.1810 |
|  | ASV496 | -0.156 | 0.047 | -3.329 | 0.0009 | 0.0009 | *Terrisporobacter* | 0.1804 |
|  | ASV479 | -0.184 | 0.054 | -3.441 | 0.0006 | 0.0006 | *Eubacterium hallii* group | 0.1155 |
|  | ASV718 | -0.211 | 0.055 | -3.875 | 0.0001 | 0.0001 | *Anaeromyxobacter* | 0.1124 |
|  | ASV541 | 0.230 | 0.039 | 5.959 | 0.0000 | 0.0000 | *Erysipelatoclostridium* | 0.1121 |
|  | ASV583 | 0.250 | 0.046 | 5.454 | 0.0000 | 0.0000 | *WCHB1-41* | 0.0000 |
| Tulathromycin Group | ASV398 | 0.158 | 0.053 | 2.963 | 0.0030 | 0.0030 | *Lactobacillus* | 15.1346 |
|  | ASV890 | -0.223 | 0.106 | -2.107 | 0.0351 | 0.0351 | *Mycoplasma* | 6.9731 |
|  | ASV872 | 0.121 | 0.052 | 2.333 | 0.0197 | 0.0197 | *Treponema 2* | 0.5043 |
|  | ASV348 | 0.142 | 0.053 | 2.675 | 0.0075 | 0.0075 | *Helicobacter* | 0.4277 |
|  | ASV175 | 0.645 | 0.116 | 5.572 | 0.0000 | 0.0000 | *Butyricimonas* | 0.2525 |
|  | ASV97 | -0.127 | 0.052 | -2.434 | 0.0149 | 0.0149 | *Rothia* | 0.2021 |
|  | ASV373 | -0.245 | 0.125 | -1.963 | 0.0497 | 0.0497 | *Jeotgalicoccus* | 0.1957 |
|  | ASV392 | 0.438 | 0.128 | 3.414 | 0.0006 | 0.0006 | *Jeotgalibaca* | 0.1692 |
|  | ASV673 | -0.123 | 0.051 | -2.398 | 0.0165 | 0.0165 | *Xanthobacteraceae** | 0.0444 |
| Florfenicol   Group | ASV679 | -1.291 | 0.129 | -10.032 | 0.0000 | 0.0000 | *Paracoccus* | 5.5131 |
|  | ASV408 | 0.096 | 0.048 | 2.009 | 0.0445 | 0.0445 | *Clostridium sensu stricto 1* | 2.0781 |
|  | ASV484 | 0.537 | 0.126 | 4.243 | 0.0000 | 0.0000 | *Ruminococcus torques group* | 0.9461 |
|  | ASV192 | 0.399 | 0.157 | 2.543 | 0.0110 | 0.0110 | *Prevotella 2* | 0.6899 |
|  | ASV840 | -0.373 | 0.062 | -6.005 | 0.0000 | 0.0000 | *Pasteurella* | 0.6652 |
|  | ASV506 | 0.372 | 0.092 | 4.062 | 0.0000 | 0.0000 | *Fournierella* | 0.5561 |
|  | ASV197 | 0.199 | 0.059 | 3.349 | 0.0008 | 0.0008 | *Prevotellaceae** | 0.5436 |
|  | ASV396 | 0.095 | 0.048 | 1.990 | 0.0466 | 0.0466 | *Enterococcus* | 0.5064 |
|  | ASV632 | 0.342 | 0.161 | 2.126 | 0.0335 | 0.0335 | *Caulobacter* | 0.2487 |
|  | ASV225 | 0.457 | 0.169 | 2.703 | 0.0069 | 0.0069 | *Sediminibacterium* | 0.2363 |
|  | ASV483 | -0.176 | 0.089 | -1.979 | 0.0478 | 0.0478 | *Ruminococcus gnavus group* | 0.2232 |
|  | ASV470 | 0.175 | 0.087 | 2.012 | 0.0442 | 0.0442 | *Lachnospiraceae XPB1014 group* | 0.1352 |
|  | ASV327 | -0.200 | 0.100 | -1.987 | 0.0469 | 0.0469 | *Gastranaerophilales* | 0.1206 |
| * ASV not classified at Genus level | | | | | | | | |

| **Supplementary Table 2.** Information of the BRD-affected animals NP sample collection**.** | | | | |
| --- | --- | --- | --- | --- |
|  | **Date of Study** | | | |
| **Calf** | **d0** | **d1** | **d5** | **d10** |
| **1** | 9 | NA | NA | NA |
| **9** | 11 | 12 | 16 | 21 |
| **15** | 17 | 18 | 22 | 27 |
| **18** | 0 | 1 | 5 | 10 |
| **19** | 2 | 3 | 7 | 12 |
| **21** | 8 | 9 | 13 | 18 |
| **23** | 0 | 1 | 5 | 10 |
| **28** | 11 | 12 | 16 | 21 |
| **29** | 7 | 8 | 12 | 17 |
| **30** | 2 | 3 | 7 | 12 |
| NA = No sample collected due to mortality | | | | |
